# The first nationwide multicenter study of *Acinetobacter baumannii* recovered in Serbia: emergence of OXA-72, OXA-23 and NDM-1-producing isolates

**DOI:** 10.1186/s13756-020-00769-8

**Published:** 2020-07-06

**Authors:** Bojana Lukovic, Ina Gajic, Ivica Dimkic, Dusan Kekic, Sanja Zornic, Tatjana Pozder, Svetlana Radisavljevic, Nataša Opavski, Milan Kojic, Lazar Ranin

**Affiliations:** 1 Institute of Microbiology and Immunology, Medical Faculty, University of Belgrade, Doktora Subotica starijeg 1, Belgrade, 11000 Serbia; 2 Faculty of Biology, University of Belgrade, Belgrade, Serbia; 3 Department of Microbiology, Clinical Center Kragujevac, Kragujevac, Serbia; 4 Department of Microbiology, General Hospital Subotica, Subotica, Serbia; 5 Department of Microbiology, General Hospital Pancevo, Pancevo, Serbia; 6 Laboratory for Molecular Microbiology, Institute of Molecular Genetics and Genetic Engineering, University of Belgrade, Belgrade, Serbia

**Keywords:** *Acinetobacter baumannii*, CRAB, *bla*_OXA23_, *bla*_OXA72_, *bla*_NDM-1_, ST492, ST636

## Abstract

**Background:**

The worldwide emergence and clonal spread of carbapenem-resistant *Acinetobacter baumannii* (CRAB) is of great concern. The aim of this nationwide study was to investigate the prevalence of CRAB isolates in Serbia and to characterize underlying resistance mechanisms and their genetic relatedness.

**Methods:**

Non-redundant clinical samples obtained from hospitalized patients throughout Serbia were included in the prospective, observational, multicenter study conducted from January to June 2018. Samples were initially screened for the presence of *Acinetobacter baumannii-calcoaceticus* (Acb) complex using conventional bacteriological techniques. Acb complexes recovered from clinical samples obtained from inpatients with confirmed bacterial infections were further evaluated for the presence of *A. baumannii*. Identification to the species level was done by the detection of the *bla*_OXA-51_ gene and *rpoB* gene sequence analysis. Susceptibility testing was done by disk diffusion and broth microdilution method. CRAB isolates were tested for the presence of acquired carbapenemases *(bla*_OXA-24-like_, *bla*_OXA-23-like,_*bla*_OXA-58-like_, *bla*_OXA-143-like_, *bla*_IMP_, *bla*_VIM_, *bla*_GIM_, *bla*_SPM_, *bla*_SIM_, *bla*_NDM_) by PCR. Clonal relatedness was assessed by pulsed-field gel electrophoresis (PFGE) and multilocus sequence typing (MLST).

**Results:**

Acb complex was isolated in 280 out of 2401 clinical samples (11.6%). Overall, *A. baumannii* was identified in 237 out of 280 Acb complex (84.6%). CRAB prevalence was found to be 93.7% (237/222). The MIC_50_/MIC_90_ for imipenem and meropenem were 8/> 32 μg/mL and 16/> 32 μg/mL, respectively. Although susceptibility was high for colistin (95.7%; *n* = 227) and tigecycline (75.1%; *n* = 178), ten isolates (4.3%) were classified as pandrug-resistant. The following carbapenemases-encoding genes were found: 98 (44.2%) *bla*_OXA-24-like_, 76 (34.5%) *bla*_OXA-23-like_, and 7 (3.2%) *bla*_NDM-1_. PFGE analysis revealed six different clusters. MLST analysis identified three STs: ST2 (*n* = 13), ST492 (*n* = 14), and ST636 (*n* = 10). Obtained results evaluated that circulating CRAB clones in Serbia were as follows: *bla*_OXA66_/*bla*_OXA23_/ST2 (32.4%), *bla*_OXA66_/*bla*_OXA23_/*bla*_OXA72_/ST2 (2.7%), *bla*_OXA66_/*bla*_OXA72_/ST492 (37.8%), and *bla*_OXA66_/*bla*_OXA72_/ST636 (27.1%).

**Conclusion:**

This study revealed extremely high proportions of carbapenem resistance among *A. baumannii* clinical isolates due to the emergence of *bla*_OXA-72_, *bla*_OXA-23_, and *bla*_NDM-1_ genes among CRAB isolates in Serbia and their clonal propagation.

## Background

In the recent years *Acinetobacter baumannii* has rapidly emerged as significant opportunistic pathogen and major cause of morbidity and mortality in the healthcare settings. The abilities of the organism to long-term survive in the hospital environment, rapidly develop antibiotic resistance and spread clonally potentiate the persistence and transmission of the species in healthcare settings. Consequently, *A. baumannii* is able to cause a spectrum of severe nosocomial infections such as ventilator-associated pneumonia, wound infections, bloodstream infections, meningitis, and urinary tract infections. The high risk populations are debilitating patients, those admitted to the intensive care units (ICUs), attached to indwelling foreign devices or mechanically ventilated [1, 2].

*A. baumannii* has a numerous inherent and acquired resistance mechanisms which dramatically reduce the existing therapeutic options [3]. Consequently, *A. baumannii* is one of the ESKAPE organisms (*Enterococcus faecium, Staphylococcus aureus, Klebsiella pneumoniae, Acinetobacter baumannii, Pseudomonas aeruginosa*, and *Enterobacter* species), a group of clinically important pathogens that have the potential for substantial antimicrobial resistance [4]. Carbapenems used to be the mainstay for the treatment of nosocomial infections caused by *A. baumannii* [3]. However, the resistance to carbapenems has rapidly emerged and resulted in the worldwide dissemination of carbapenem-resistant *A. baumannii* (CRAB) strains, which usually exhibits a multidrug-resistant (MDR) phenotype, raising serious concerns about available treatment options [5, 6]. In addition, CRAB is one of the critical-priority pathogens on the World Health Organization priority list of antibiotic-resistant bacteria for effective drug development [7]. Moreover, in critically ill patients in the ICUs infected with MDR *A. baumannii*, there is a substantial rise in patient mortality rates [8]. According to the most recent data from Centers for Disease Control and Prevention CRAB is listed as urgent threat in the United States with 8.500 cases and 700 deaths in hospital settings in 2017 [9].

Carbapenem resistance in *Acinetobacter* is mostly associated with the production of carbapenem-hydrolyzing enzymes, Ambler class D *β-lactamases* or oxacillinases (OXAs) [10]. Although the OXAs weakly hydrolyze carbapenems, they can confer higher resistance when *bla*_OXA_ genes are overexpressed using a strong promoter with mobile insertion elements such as IS*Aba1* [11]. Less frequent, but much powerful carbapenem-hydrolyzing enzymes in *A. baumannii* belong to class B *β*-*lactamases* or metallo-*β*-*lactamases* (MBLs) [10, 12].

There are numerous reports about the dissemination of CRAB strains, carriers of the OXA and MBL genes, from different geographical areas around the world [5, 12, 13]. Molecular typing of the CRAB strains from various European hospitals has shown the emergence of three successful clones originally named European clones I to III, which were renamed as international clones (ICs) I to III, after been identified worldwide [14]. In Serbia, there is no data on molecular epidemiology of CRAB in adult patients. So far, there is just one study, reporting the distribution of carbapenemase encoding genes among clinical isolates of *A. baumannii* from a single pediatric hospital [15]. However, there are several reports on OXA and MBL-positive CRAB strains, recovered from patients migrating from Serbia to Western Europe [16, 17].

Therefore, the aims of this nationwide multicenter study were: *i*) to evaluate the prevalence and antimicrobial resistance patterns of *A. baumannii* circulating in Serbia, *ii*) to determine the prevalence of OXAs and MBLs among CRAB isolates, and *iii*) to assess their genetic relatedness and identify disseminated CRAB clones.

## Methods

### Study design and setting

The study was designed as a prospective, observational multicenter study. Serbia is Southeastern European country with roughly seven million inhabitants. It could be arbitrarily divided into three regions: Belgrade (the capital, two million), South Serbia (three million) and Vojvodina (two million). In the present study, regional microbiological laboratories from seven cities across Serbia evaluated 2401 randomly selected clinical specimens recovered from inpatients with confirmed bacterial infections admitted at nine hospitals throughout Serbia during the period January–June 2018. Microbiological laboratories from each region of the country evaluated the following number of hospital specimens: Vojvodina, *n* = 800 (General hospital Subotica, General hospital Pancevo, General hospital Sombor and Institute for pulmonary diseases of Vojvodina), Belgrade, *n* = 801 (University hospital medical center Bezanijska kosa, University hospital center dr Dragisa Misovic, Institute for cardiovascular diseases Dedinje), and Southern Serbia *n* = 800 (Clinical center Kragujevac, Clinical center Nis). All isolated *Acinetobacter calcoaceticus–baumannii* (Acb) complexes were sent to the coordinating laboratory at the Institute of Microbiology and Immunology of Medical Faculty University in Belgrade for further investigation. The inclusion criteria was the isolation of the Acb complex from non-redundant clinical samples (one per infected patient) obtained during the routine laboratory work. Infection was diagnosed using corresponding clinical and laboratory criteria. The exclusion criteria were detection of the non-Acb complex microbial agents as a possible source of infection and/or isolation of Acb complex without any clinical manifestations related to infection or without positive laboratory inflammatory markers. We addressed the potential bias of misdiagnosis of infectious disease by having the diagnostic criteria validated by an independent physician. This physician was blinded in terms of medical records.

### Bacterial isolation and identification

Bacterial isolation and identification of Acb complex was done during the routine work in regional clinical laboratories by VITEK®2 system (bioMérieux, Marcy-l’Étoile, France) and conventional bacteriological techniques, such as colony appearance on sheep blood agar and MacConkey agar, Gram staining, motility test and biochemical characteristics.

All isolated Acb complexes together with the following data: type of specimen, ward of admission, patients’ age, gender, clinical diagnosis, underlying conditions and other risk factors were sent to the coordinating laboratory for further investigation. All patients were de-identified in regional clinical laboratories and re-coded, before the bacterial strains were sent to the coordinating laboratory. The study was approved by the ethical committee of the Medical Faculty, University of Belgrade (1550/II-4).

Species identification was done in the coordinating laboratory. The DNA from an overnight culture on Columbia blood agar was extracted using a QIAamp DNA Mini Kit (QIAGEN GmbH, Hilden, Germany) according to the manufacturer’s instructions. All isolates of Acb complex were initially screened for the presence of *A. baumannii* by PCR detection of the intrinsic *bla*_OXA-51_ gene, as previously reported [18]. The primers are shown in Table 1. The species identification was then confirmed by *rpoB* gene sequence analysis, as previously described [23]. All identified *A. baumannii* strains were stored at − 80 °C until further analysis.

**Table 1:** Primers used in this study

Primer	Sequence	Amplicon size (bp)	Reference
OXA-51-likeF	5′-TAATGCTTTGATCGGCCTTG-3’	353	[18]
OXA-51-likeR	5′-TGGATTGCACTTCATCTTGG-3’		
OXA-143-likeF	5′-TGGCACTTTCAGCAGTTCCT-3’	149	[19]
OXA-143-likeR	5′-TAATCTTGAGGGGGCCAACC-3’		
IMP-F	5′-GGAATAGAGTGGCTTAAYTCTC-3’	232	[20]
IMP-R	5′-GGTTTAAYAAAACAACCACC-3’		
VIM-F	5′-GATGGTGTTTGGTCGCATA-3’	390	[20]
VIM-R	5′-CGAATGCGCAGCACCAG-3’		
GIM-F	5′-TCGACACACCTTGGTCTGAA-3’	477	[20]
GIM-R	5′-AACTTCCAACTTTGCCATGC-3’		
SPM-F	5′-AAAATCTGGGTACGCAAACG-3’	271	[20]
SPM-R	5′-ACATTATCCGCTGGAACAGG-3’		
SIM-F	5′-TACAAGGGATTCGGCATCG-3’	570	[20]
SIM-R	5′-TAATGGCCTGTTCCCATGTG-3’		
NDM-F	5′-GGTTTGGCGATCTGGTTTTC-3’	621	[20]
NDM-R	5′-CGGAATGGCTCATCACGATC-3’		
OXA-23-likeF	5′-GATCGGATTGGAGAACCAGA-3’	501	[21]
OXA-23-likeR	5′-ATTTCTGACCGCATTTCCAT-3’		
OXA-24-likeF	5′-GGTTAGTTGGCCCCCTTAAA-3’	246	[21]
OXA-24-likeR	5′-AGTTGAGCGAAAAGGGGATT-3’		
OXA-58-likeF	5′-AAGTATTGGGGCTTGTGCTG-3’	599	[21]
OXA-58-likeR	5′-CCCCTCTGCGCTCTACATAC-3’		
IS*Aba1*-F	5′-CACGAATGCAGAAGTTG-3’	549	[22]
IS*Aba1*-R	5′-CGACGAATACTATGACAC-3’		

### Antimicrobial susceptibility testing

Antimicrobial susceptibility of *A. baumannii* to ampicillin-sulbactam (AMS), piperacillin-tazobactam (TZP), ceftazidime (CAZ), cefepime (FEP), meropenem (MER), imipenem (IMP), gentamicin (GEN), amikacin (AK), tobramycin (TOB), tetracycline (TET), ciprofloxacin (CIP), levofloxacin (LEV), and trimethoprim-sulfamethoxazole (TSX) was determined by disk-diffusion assay (Bio-Rad, UK), following the Clinical and Laboratory Standards Institute (CLSI) guidelines [24]. Minimum inhibitory concentrations (MICs) for colistin (COL), IMP and MER were evaluated by ComASP Colistin (Liofilchem, Italy) and Gradient strip test (Liofilchem, Italy), respectively. MICs for tigecycline (TYG) were assessed by broth microdilution method with Mueller-Hinton broth (Bio-Rad, UK), following the CLSI guidelines [24]. Susceptibility categories were interpreted according to the CLSI criteria [24] for all antibiotics except for TYG which was interpreted according to the European Committee on Antimicrobial Susceptibility Testing (EUCAST) breakpoints for Enterobacterales (S ≤ 1 μg/mL; R > 2 μg/mL), since the breakpoints for TYG susceptibility against *A. baumannii* have not been determined yet [25]. *Escherichia coli* ATCC 25922 and *Pseudomonas aeruginosa* ATCC 27853 were used as quality control strains. The isolates of *A. baumannii* were classified as follows: multidrug-resistant (MDR) [resistant to at least one agent in three or more antimicrobial categories], extensively drug-resistant (XDR) [resistant to at least one agent in all, but two or fewer antimicrobial categories] and pandrug-resistant (PDR) [resistant to all agents in all antimicrobial categories tested] [26]. Additionally, IMP resistant *A. baumannii* isolates were selected for the phenotypic detection of MBL production by imipenem-EDTA combined disk test [27].

### Detection of carbapenemase encoding genes and IS*Aba1* element

All CRAB isolates were screened for the presence of *bla*_OXA-23-like_, *bla*_OXA-24-like_, *bla*_OXA-58-like_, *bla*_OXA-143-like_, *bla*_IMP_, *bla*_VIM_, *bla*_GIM_, *bla*_SPM_, *bla*_SIM_ and *bla*_NDM_ with primers published elsewhere (Table 1) [19–21]. Multiplex PCRs were performed with separate groups of 4–6 primers and following the same thermal cycling conditions: initial denaturation at 94 °C for 10 min and 36 cycles of amplification consisting of 30 s at 94 °C, 40 s at 52 °C, and 50 s at 72 °C, with 5 min at 72 °C for the final extension. Deionized distilled water was used as the negative control and isolates whose sequences were previously confirmed were used as the positive control. IS*Aba1* was detected performing uniplex PCR, as previously reported [22]. The primers are shown in Table 1. Randomly selected OXA and all MBL amplicons of CRAB isolates were purified using the QIAquick PCR Purification Kit (QIAGEN GmbH, Hilden, Germany) following manufacturer’s instructions and sequenced using conventional Sanger sequencing method. The obtained sequences were searched for homology with sequenced genes in the GenBank database using the National Center for Biotechnology Information’s BLAST search program 2.7.0 for nucleotides (http://www.ncbi.nlm.nih.gov). All gene sequences (manually checked) and reference strain sequences from GenBank database were aligned using CLUSTAL W implemented in BioEdit ver. 7.1.3 software.

### Molecular typing

#### Pulsed-field gel electrophoresis (PFGE) analysis

PFGE included 60 CRAB isolates randomly selected from all participating hospitals, with respect to the specimen types and gene content. PFGE was performed with a 2015 Pulsafor unit (LKB Instruments, Broma, Sweden), as previously described [28]. DNA restriction was done with ApaI enzyme (Thermo Scientific, Lithuania). The Lambda Ladder 48.5–727.5 kb PFG Marker (New England Biolabs, US) was used as DNA size marker. The stained gels were scanned using the Diversity Database software image-capturing system (Bio-Rad Laboratories Ltd., UK). The Dice coefficient was used to calculate similarities of the banding patterns with a tolerance of 1.5%, and the unweighted-pair group method using average linkages (UPGMA) was used for cluster analysis with BioNumerics software, version 4.0 (Applied Maths, St-Martens-Latem, Belgium). The isolates with more than 80% similarity in their DNA patterns were defined as genetically related and part of the same cluster.

#### Multilocus sequence typing (MLST) analysis

At least 50% of the CRAB isolates from each PFGE cluster and at least one isolate from each participating hospital, endowed with different *bla*_OXA_ gene content were selected for MLST analysis. Overall, 37 isolates were typed by MLST under the Pasteur MLST scheme which involved PCR amplification, purification and sequencing of seven housekeeping genes (*fusA*, *gltA*, *pyrG*, *recA*, *cpn60*, *rpoB*, and *rplB*), as indicated previously [29]. Sequence types (STs) were assigned using the same scheme from the MLST website (http://pubmlst.org/abaumannii/).

### Statistical analysis

Statistical analysis was done using SPSS version 14.0 (Chicago, Illinois USA). Statistical significance was assessed using the χ^2^ test or Fisher’s exact test, as appropriate. A *P* ≤ 0.05 value was considered to be significant.

## Results

### Bacterial isolates

**Fig. 1:**
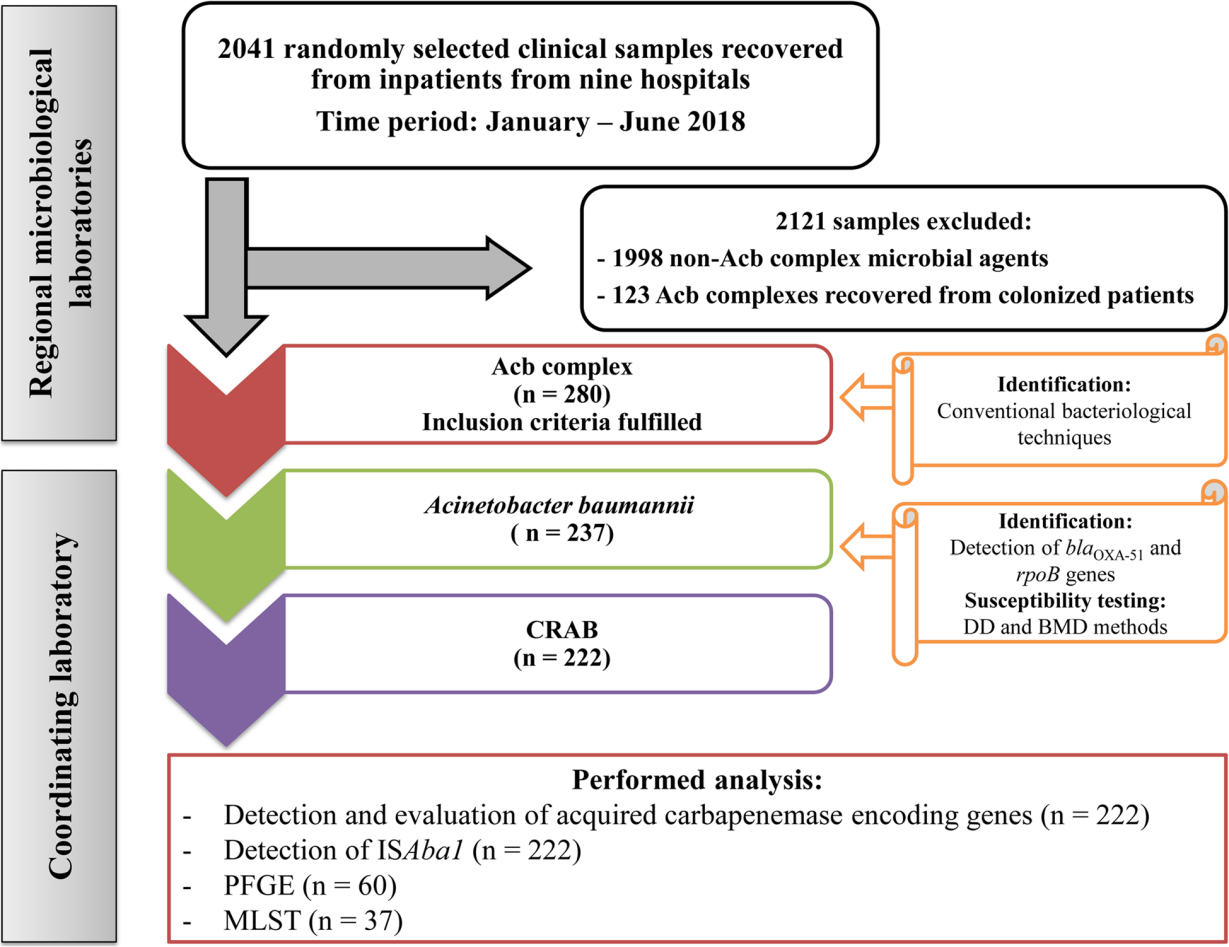
Flowchart of the process of Acb complex inclusion and strain selection for further molecular evaluation. Inclusion criteria was isolation of the Acb complex from non-redundant clinical samples (one per infected patient) obtained during the routine laboratory work. Acb complex - *Acinetobacter calcoaceticus–baumannii* complex; *A. baumannii* - *Acinetobacter baumannii*; BMD - Broth microdilution; CRAB - Carbapenem-resistant *A. baumannii;* DD - Disk diffusion; MLST - Multilocus sequence typing; PFGE - Pulsed-field gel electrophoresis

During the study period, 2401 clinical samples were screened for inclusion (Fig. 1). A total of 280 (11.6%) isolates of Acb complex recovered from clinical samples fulfilled inclusion criteria. *A. baumannii* was identified in 237 out of 280 isolates of Acb complex (84.6%). Overall, the prevalence of the infections caused by *A. baumannii* were as follows: sepsis (*n* = 28/351; 7.9%), lower respiratory tract infections (*n* = 89/319; 27.9%), skin and soft tissue infections (*n* = 87/511; 17%), meningitis (*n* = 1/34; 2.9%), central venous catheter-related infections (*n* = 14/94; 14.9%), and urinary tract infections (*n* = 18/1092; 1.6%). Most of the *A. baumannii* isolates were obtained from the ICU (39.6%). Bacterial isolates were predominantly recovered from lower respiratory tract specimens (37.6%), wound exudates (36.7%) and blood samples (11.8%). Majority of the patients were elderly men (median age 66; within the range 14–87). Diabetes mellitus (23.2%) and surgical procedures (46.8%) were the most commonly reported underlying conditions. Detail information concerning specimen types, patient characteristics, underlying conditions, and risk factors are listed in Table 2.

**Table 2:** Demographic and clinical characteristics of *Acinetobacter baumannii-*infected study patients

Characteristics	Patients No. (%)
**Gender**	
Male	148 (62.5)
Female	89 (37.5)
**Admission ward**	
Intensive care unit	94 (39.6)
Thoracic surgery	10 (4.3)
Orthopedic surgery	14 (5.9)
Plastic surgery	16 (6.7)
Vascular surgery	15 (6.4)
Neurosurgery	9 (3.8)
General surgery	12 (5.1)
Urology surgery	7 (2.9)
Internal wards	60 (25.3)
**Type of specimen**	
Tracheal aspirate	61 (25.7)
Bronchial aspirate	19 (8.1)
Sputum	9 (3.8)
Wound exudate	87 (36.7)
Blood	28 (11.8)
Central venous catheter tip	14 (5.9)
Urine	18 (7.6)
Cerebrospinal fluid	1 (0.4)
**Comorbidity**	
Diabetes mellitus	55 (23.2)
Hypertension	53 (22.4)
Heart insufficiency	46 (19.4)
Cerebrovascular disease	42 (17.7)
Chronic venous insufficiency	35 (14.7)
Chronic obstructive lung disease	25 (10.5)
Renal failure	21 (8.9)
Chronic liver disease	1 (0.4)
Neuromuscular disorders	0
Psychiatric disorders	0
Malignancy	19 (8.0)
Immunological disorders	1 (0.4)
Hematologic disorders	0
Polytrauma	13 (5.5)
Severe burns	9 (3.8)
**Invasive procedures**	
Any surgical procedure	111 (46.8)
Mechanical ventilation	68 (28.7)
Tracheostomy	11 (4.6)
Central venous catheter	46 (19.4)
Urinary catheter	68 (28.7)

### Antimicrobial resistance

**Fig. 2:**
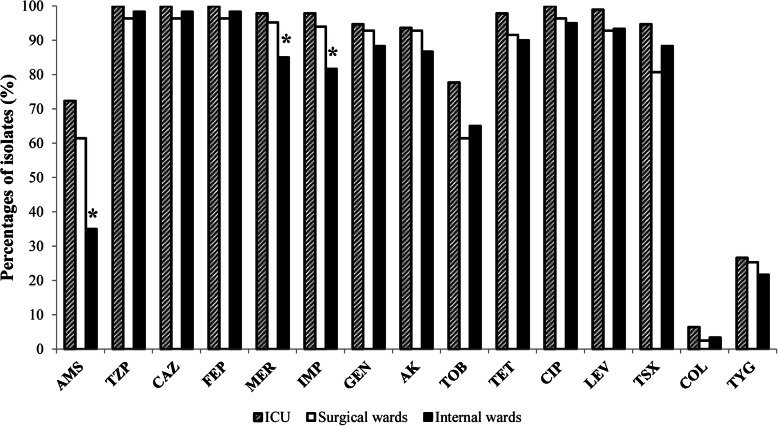
Antimicrobial resistance of *A. baumannii* in different hospital wards. AMS – ampicillin-sulbactam; TZP - piperacillin-tazobactam; CAZ - ceftazidime; FEP - cefepime; MER - meropenem; IMP - imipenem; GEN - gentamicin; AK - amikacin; TOB - tobramycin; TET - tetracycline; CIP - ciprofloxacin; LEV - levofloxacin; TSX - trimethoprim-sulfamethoxazole; COL - colistin; TYG - tigecycline.*P < 0.05

The antibiotic resistance patterns of 237 *A. baumannii* isolates are shown in Table 3. CRAB prevalence was found to be 93.7%. Three isolates were resistant to MER and sensitive to IMI, while 219 (92.4%) were resistant to both IMP and MER. Fifteen isolates (6.3%) were fully susceptible to carbapenems. The MICs of the tested carbapenems were 2–256 μg/mL. The MIC_50_/MIC_90_ for IMP and MER were 8/> 32 μg/mL and 16/> 32 μg/mL, respectively. Carbapenem resistance rate was higher in non-invasive (94.9%) than in invasive (92.1%) *A. baumannii* isolates. However, statistical significance was not observed (*P* > 0.05). Moderate resistance rates were observed for AMS (59.1%) and TOB (68.8%). The drugs for which susceptibility was highest were the last-resort drugs, COL (95.7%) and TYG (75.1%). Thus, the MICs for COL ranged from < 0.25 to 4 μg/mL with MIC_50_/MIC_90_ values < 0.25/1 μg/mL, while the MICs for TYG ranged from 0.125–16 μg/mL with MIC_50_/MIC_90_ values 2/8 μg/mL. Resistance rates for all tested antibiotics were higher in ICU compared to other wards (Fig. 2), with the statistical significance for AMS, IMP and MER (*P* < 0.05). Although geographical differences of the proportion of CRAB isolates were detected (Vojvodina-91.5%; Belgrade-87.5%; Southern Serbia-96.3%), statistical significance was not observed (*P* > 0.05). Overall, CRAB isolates exhibited higher resistance rates to all antimicrobials as compared to carbapenems-susceptible *A. baumannii* (Table 3), with the exception of TYG and COL. Two hundred and three (85.6%) isolates were considered MDR. Ninety-five isolates (40%) displayed an XDR phenotype. Furthermore, ten isolates (4.3%) were classified as PDR. MDR, XDR and PDR were more prevalent in the ICU than in other hospital units. The phenotypic method revealed MBL production in 5 (2.23%) out of 222 CRAB strains.

**Table 3:** Antimicrobial resistance of the *A. baumannii* isolates resistant and susceptible to carbapenems

Antimicrobial agent	Overall resistance (***n*** = 237) No. (%)	Carbapenem-resistant ***A. baumannii*** (***n*** = 222) No. (%)	Carbapenem-susceptibile***A. baumannii*** (***n*** = 15) No. (%)	***P*** value
Ampicillin-sulbactam	140 (59.1%)	136 (61.3)	4 (26.7)	*P* = 0.008
Piperacillin-tazobactam	233 (98.3%)	222 (100)	11 (73.3)	*P* < 0.001
Ceftazidime	233 (98.3%)	222 (100)	11 (73.3)	*P* < 0.001
Cefepime	233 (98.3%)	222 (100)	11 (73.3)	*P* < 0.001
Imipenem	219 (92.4%)	219 (98.6)	0 (0)	*P* < 0.001
Meropenem	222 (93.7%)	222 (100)	0 (0)	*P* < 0.001
Amikacin	217 (91.6%)	209 (94.1)	8 (53.3)	*P* < 0.001
Gentamicin	220 (92.8%)	210 (94.6)	10 (66.7)	*P* < 0.001
Tobramycin	163 (68.8%)	159 (71.6)	4 (26.7)	*P* = 0.000
Ciprofloxacin	231 (97.5%)	221 (99.5)	10 (66.7)	*P* < 0.001
Levofloxacin	226 (95.4%)	217 (97.7)	9 (60)	*P* < 0.001
Trimethoprim-sulfamethoxazole	209 (88.2%)	202 (91)	6 (40)	*P* < 0.001
Tetracycline	222 (93.7%)	214 (96.4)	8 (53.3)	*P* < 0.001
Tigecycline	59 (24.9%)	58 (26.1)	1 (6.7)	*P* = 0.092
Colistin	10 (4.3%)	10 (4.5)	0 (0)	*P* = 0.401

### Detection of carbapenemase encoding genes and IS*Aba1* element in *A. baumannii*

A total of 237 *A. baumannii* isolates harbored the naturally occurring *bla*_OXA-51_-like gene. Out of 222 CRAB isolates, 98 isolates (44.2%) carried *bla*_OXA-24-like_ and 76 isolates (34.5%) carried *bla*_OXA-23-like_ genes. Two isolates had both *bla*_OXA-23_ and *bla*_OXA-24_ genes, simultaneously. However, *bla*_OXA-58-like_ and *bla*_OXA-143-like_ genes were not detected in the tested population. Overall, IS*Aba1* was present in 161 (71.8%) CRAB strains. The co-existence of IS*Aba1*/*bla*_OXA-51-like_, IS*Aba1*/*bla*_OXA-24-like_, IS*Aba1*/*bla*_OXA-23-like_ and *bla*_OXA-51_/*bla*_OXA-23_/*bla*_OXA-24_ genes was detected in 16.5, 28.1, 26.7, and 0.5% of CRAB strains, respectively. The *bla*_NDM_ was the only MBL gene detected in the study (*n* = 7). Sequencing of the *bla*_NDM_ genes in all 7 isolates revealed the presence of the *bla*_NDM-1_ variant. Furthermore, all *bla*_NDM-1_ positive *A. baumannii* were *bla*_OXA-24_ positive isolates. None of the isolates carried *bla*_IMP_, *bla*_VIM_, *bla*_GIM_, *bla*_SPM_ and *bla*_SIM_ genes. There were no substantial differences between the hospitals and regions regarding the proportion of isolates carrying different acquired OXA genes. However, *bla*_NDM-1_ positive isolates were detected only in two cities: Belgrade (the capital) and Nis (the city in Southern Serbia).

### Molecular typing

**Fig. 3:**
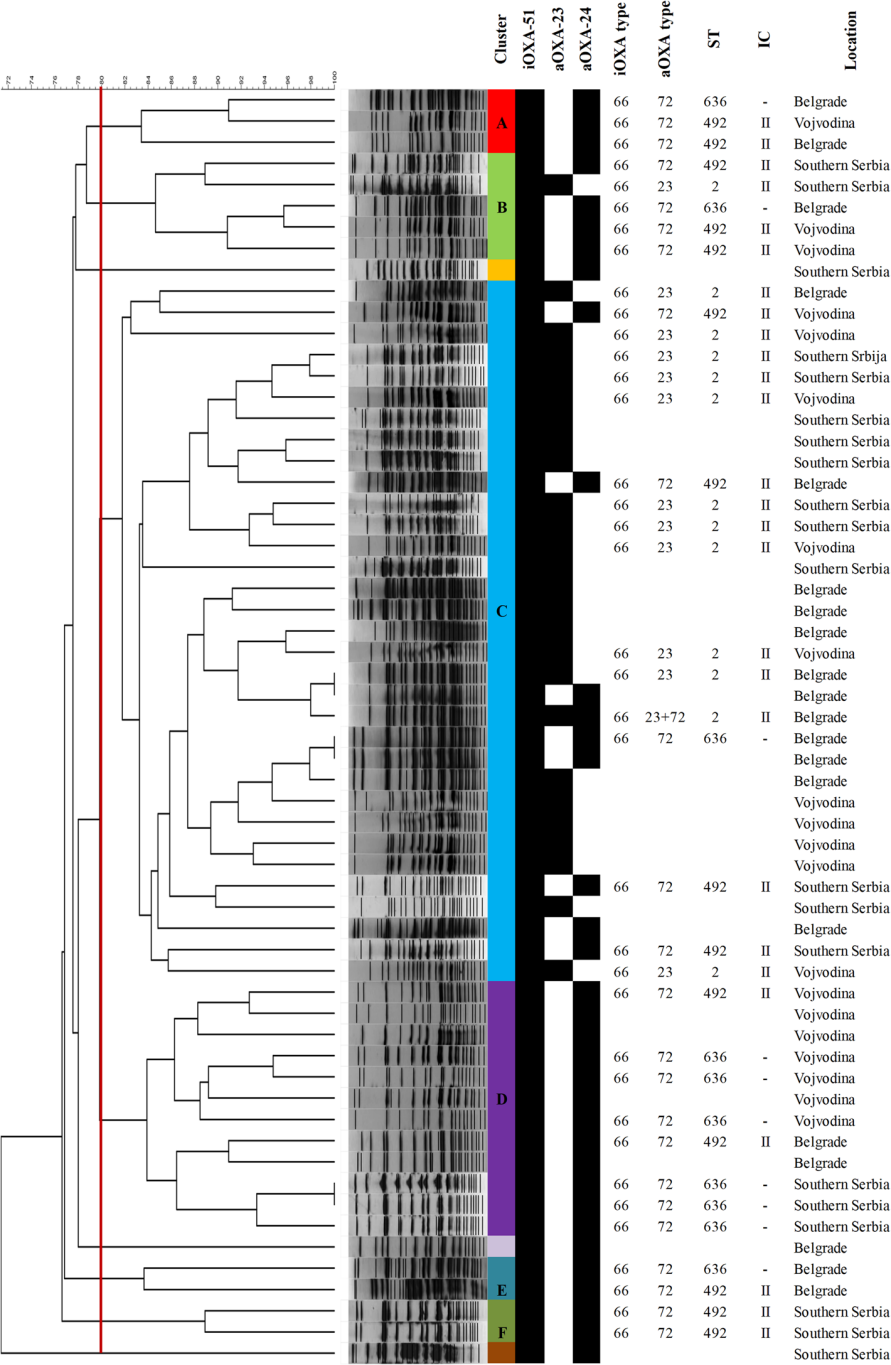
Dendrogram of the pulsed-field gel electrophoresis patterns of the *A. baumannii* strains isolated from nine regional hospitals in Serbia showing clusters (A-F); carriage of intrinsic (iOXA) and acquired *bla*_OXA_ variants (aOXA); sequence type (ST) and corresponding international clone (IC), and hospital location

The results of PFGE and MLST analysis of the tested CRABs, along with the information on the hospital locations, intrinsic and acquired OXA gene content are summarized in Fig. 3.

The clonal relatedness of 60 CRAB isolates obtained from all participating hospitals was initially studied by PFGE. The similarity of the tested CRAB isolates ranged from 70 to 99%. Overall, PFGE analysis revealed six different clusters (A-F) and three singletons (Fig. 3). Largest clusters B, C and D which contained 5 (8.3%), 33 (55%), and 12 (20%) isolates, respectively, were comprised of CRAB isolates from Vojvodina, Belgrade and Southern Serbia. Isolates in cluster B and C were associated with the carriage of both *bla*_OXA-24-like_ and *bla*_OXA-23-like_ carbapenemases. Isolates of the remaining four clusters [cluster A (*n* = 3, 5%), cluster D (*n* = 12, 20%), cluster E (*n* = 2, 3.3%), and cluster F (*n* = 2, 3.3%)] were more homogenous and carried only *bla*_OXA-24-like_ genes. Based on the PFGE clustering, OXA genes content, and hospital origin, out of 60 CRAB isolates analyzed by PFGE, 37 were selected for further characterization by MLST and OXA gene sequencing.

MLST assigned the analyzed CRAB isolates to 3 STs: ST2 (*n* = 13), ST492 (*n* = 14), and ST636 (*n* = 10). ST2 and ST492 (a single locus variant of ST2) belonged to ICII. ST 636 (a triple locus variant of ST2) was a singleton, not categorized in any IC. The *bla*_OXA-23_ positive isolates, were all assigned to ST2, *bla*_OXA-24_ positive belonged to ST492 and ST636, while *bla*_NDM-1_ positive strains were identified as ST2 or ST492. All three STs were evenly distributed in Vojvodina, Belgrade and Southern Serbia. Sequencing of the acquired class D OXA genes in all selected isolates revealed the presence of the *bla*_OXA-72_ variant in all *bla*_OXA-24-like_ positive isolates and *bla*_OXA-23_ allele in all *bla*_OXA-23-like_ positive isolates. Sequencing of the intrinsic class D OXA gene identified *bla*_OXA-66_ gene in all 37 isolates tested (Fig. 3). Therefore, detected clones of CRAB isolates circulating in Serbia were as follows: *bla*_OXA66_/*bla*_OXA23_/ST2 (32.4%), *bla*_OXA66_/*bla*_OXA23_/*bla*_OXA72_/ST2 (2.7%), *bla*_OXA66_/*bla*_OXA72_/ST492 (37.8%), and *bla*_OXA66_/*bla*_OXA72_/ST636(27.1%).

## Discussion

*A. baumannii* poses a significant challenge to clinicians due to the increased incidence of hospital-acquired infections and its emergence of drug-resistance. This is the first Serbian nationwide multicenter study, providing data about the carbapenem resistance and clonality assessments of *A. baumannii* recovered from different hospitals throughout the country.

As expected, the vast majority of the Acb complexes tested in the present study was identified as *A*. *baumannii* (84.6%). Indeed, *A. baumannii* is the species of Acb complex most frequently involved in nosocomial outbreaks or sporadic infections [13]. Results of this study indicated that the prevalence of bloodstream infections caused by *A. baumannii* in Serbia (7.9%) is comparable to those found in some African and Asian countries (Morocco: 9.2%; India: 7.5%) [30, 31]. More than one-third of the tested *A. baumannii* recovered from clinical specimens were of respiratory origin (37.6%). The respiratory tract has already been confirmed to be the most common isolation site of *A. baumannii* [13]. Furthermore, nearly 50% of the isolated *A. baumannii* in Mexican tertiary care hospital were of respiratory origin while a 42.6% were found in Chinese university hospital, within a 3-year study period [32, 33]. Within our study, the second most common isolation site of *A. baumannii* was wound tissue (36.7%). Slightly lower isolation rates were recorded in China (burn wards: 25.2%; general surgery wards: 32.2%) [33] and in Mexico (23.2%) [32]. It is necessary to emphasize that the high rates of our *A. baumannii* isolated from wound exudates, point out the necessity of the strict following infection control measures, especially those focusing on the minimizing the transmission rate. In addition, various risk factors noticed in the present study, have already been significantly associated with infections caused by *A. baumannii*: mechanical ventilation, indwelling catheters, and admission to the ICUs [1]. In this study, the overall CRAB prevalence was higher than 90%, with IMP being a slightly more effective than MER (92.4% vs. 93.7%). This finding is similar to other reports of discordant susceptibilities to carbapenems, in which an isolate susceptible to imipenem but resistant to meropenem was reported [3, 34]. Polymyxins and TYG are the only antimicrobial options for XDR *Acinetobacter*. Our results revealed that COL was the most active antibiotic against *A. baumannii*. However, the detection of 10 COL resistant isolates in this study is worrisome. Although still uncommon, *A. baumannii* resistance to COL has been reported from different geographic regions [35–37]. Currently, for PDR *A. baumannii*, limited therapeutic options exist and are usually reflected in combination therapies of two or more antibiotics [38].

Among the tested CRAB isolates, *bla*_OXA-24_ was the most frequent carbapenemase gene (44.2%), followed by *bla*_OXA-23_ (34.5%), and *bla*_NDM-1_ (3.2%). Furthermore, all sequenced *bla*_OXA-24_ were identified as *bla*_OXA-72_, its single-amino-acid variant. In contrast to *bla*_OXA-23_, which is the most common acquired carbapenemase in *A. baumannii* worldwide [5], *bla*_OXA-72_ producing isolates have been reported in only several countries, including Croatia, Lithuania, and Brazil [39–41]. Results obtained in this research are in concordance with the study conducted in one pediatric hospital in Serbia, where *bla*_OXA-24_ and *bla*_OXA-23_ were also the most prevalent acquired OXAs among pediatric CRAB isolates [15]. Furthermore, *bla*_OXA-24_ was also dominant in Bulgaria (46.7%) and Croatia (97.1%), while *bla*_OXA-23_ is globally distributed, as well as in some regional countries such as Greece (96.9%) [5, 35, 39, 42]. However, contrary to our results, Novovic et al. reported *bla*_OXA-58_ in more than one-third of the tested CRAB isolated from children, indicating potential hospital dissemination of this clone [15]. Although, *bla*_OXA-58_ was also detected in all three aforementioned studies from Balkan region, its prevalence was less than 3% of CRABs [35, 39, 42]. Our results are in line with other studies showing international shift from *bla*_OXA-58_ towards *bla*_OXA-23_ or *bla*_OXA-24_ [32, 43–46].

The *bla*_NDM-1_ carrying *A. baumannii* has already emerged in Europe, Asia, Africa and South America [12, 44, 47, 48]. Interestingly, two reports, from France and Germany, characterized *bla*_NDM-1_ positive *A. baumannii* isolates obtained from patients repatriated to these countries from Serbia [16, 17]. In concordance with the above mentioned reports this study confirms that Serbia might be an endemic region for CRAB isolates carrying *bla*_NDM-1_ genes.

In the present evaluation, MLST results correlated well with PFGE clustering. The possibility that interhospital transmission of CRAB clones have occurred in Serbia is reinforced by the detection of the majority of the PFGE patterns almost uniformly distributed throughout the country. Nevertheless, with results obtained under the Pasteur MLST scheme few genetic lineages were shown to circulate in Serbia: ST636, as a singleton along with ICII representatives (ST2 and ST492), all carrying the *bla*_OXA-66_ allele of the *bla*_OXA-51-like_ gene. Similar to our findings, numerous reports highlighted that ICII is widely distributed in all continents, and notably in the Mediterranean area [14, 49]. Hence, various studies indicated the worldwide distribution of the *A. baumannii* ST2/ICII having *bla*_OXA-23_ gene [5]. Contrastingly to the aforementioned strain, the *A. baumannii* ST636 harboring *bla*_OXA-72_ gene was isolated sporadically, from patients in Sweden, Lebanon, and one Russian patient from Germany [50–52]. Furthermore, obtained results revealed that Serbia seems to remain the reservoir for the *A. baumannii* pertained to ST492/ICII, carrying *bla*_OXA-72_ and *bla*_NDM-1_ genes**.** To the best of our knowledge, besides this study, there is only one case report of infection caused by *bla*_OXA-72_/*bla*_NDM-1_ producing *A. baumannii*, belonging to ICII/ST492, recovered from a urine sample of a Serbian patient hospitalized in France [16].

The limitation of the present study could be the small sample size of the randomly selected isolates for MLST and sequencing of the genes encoding carbapenemases. Nonetheless, despite these limitations, this nationwide research provides an important insight into the molecular epidemiology of the *A. baumannii* circulating in this region, especially comparing with the majority of the available European reports on *A. baumannii* which are focused on one health-care facility and particular hospital outbreak. Moreover, obtained results would serve as the base for the future studies and trend assessment of infections caused by Acinetobacter. Consequently, these data can be used to support regional initiatives to enhance infection control practices and antimicrobial stewardship, by facilitating available and inexpensive tools and resources to tackle this problem effectively and systematically.

## Conclusion

In summary, this first nationwide study highlights the molecular epidemiology of the clinical isolates of *A. baumannii*. Alarming proportions of CRAB isolates are the consequences of the emergence of *bla*_OXA-72_, *bla*_OXA-23_, and *bla*_NDM-1_ among the tested isolates. Therefore, the extremely high antimicrobial resistance rates and the emergence of certain CRAB clones point out the critical need for the implementation of continuous surveillance of infections caused by *A. baumannii* and developing accurate prevention strategies in Serbia.

## Data Availability

All data generated or analysed during this study are included in this published article.

## Abbreviations

Acb complex: *Acinetobacter calcoaceticus–baumannii* complex
AK: Amikacin
AMS: Ampicillin-sulbactam
BMD: Broth microdilution
CDC: Centers for Disease Control and Prevention
CIP: Ciprofloxacin
CLSI: Clinical and Laboratory Standards Institute
COL: Colistin
CAZ: Ceftazidime
CRAB: Carbapenem-resistant *A. baumannii*
EUCAST: European Committee on Antimicrobial Susceptibility Testing
FEP: Cefepime
GEN: Gentamicin
ICs: International clones
ICUs: Intensive care units
IMP: Imipenem
LEV: Levofloxacin
MBLs: Metallo-*β*-*lactamases*
MDR: Multidrug-resistant
MER: Meropenem
MICs: Minimum inhibitory concentrations
MLST: Multilocus sequence typing
PDR: Pandrug-resistant
OXAs: Oxacillinases
PFGE: Pulsed-field gel electrophoresis
STs: Sequence types
TET: Tetracycline
TOB: Tobramycin
TSX: Trimethoprim-sulfamethoxazole
TYG: Tigecycline
TZP: Piperacillin-tazobactam
XDR: Extensively drug-resistant
